# Relationship between upward social comparison and adolescent depression: moderating roles of football participation

**DOI:** 10.3389/fpubh.2026.1946875

**Published:** 2026-10-14

**Authors:** Yi Zheng, Huarui Huang, Anzu Li, Chen Zhong, Long Zhang, Yizhou Shui

**Affiliations:** 1 School of Physical Education, Shaanxi Normal University, Xi’an, China; 2 School of Psychology, Shaanxi Normal University, Xi’an, China

**Keywords:** adolescent, depression, football, moderation effect, sport participation, upward social comparison

## Abstract

Adolescent depression is an important global public health concern. Although sport participation is generally associated with better mental health among adolescents, it remains unclear whether it moderates the association between upward social comparison and depressive symptoms and whether this effect differs across sport types. To address this gap, this study aimed to examine whether sport participation moderates the association and whether the effect differs between football participants and participants in individual sports. A total of 2,451 adolescents were recruited from 162 schools across 13 provinces in China; after excluding 16 participants, 2,435 were included in the final analyses (age, *M* = 12.36, SD = 3.42). Participants completed the Upward Social Comparison Scale, the Sports Participation Scale, and the Beck Depression Inventory-II. Using Hayes’ PROCESS macro (Models 1 and 3), this study assessed moderation while controlling for gender and school level. Upward social comparison was positively associated with depression (*b* = 0.079, *p* < 0.001), while sport participation was negatively associated with depression (*b* = −0.155, *p* < 0.001). Their interaction was significant (*b* = −0.055, *p* = 0.005), indicating that higher sport participation was associated with a weaker positive association between upward social comparison and depression. A three-way interaction was also significant (*b* = 0.163, *p* = 0.002), indicating that the moderation pattern differed by sport type. Among football participants, the positive association between upward social comparison and depression was weaker at higher levels of sport participation but remained statistically significant, whereas no significant association was observed at either level of sport participation among participants in individual sports. In conclusion, higher levels of sport participation were associated with a weaker positive association between upward social comparison and adolescent depression, and this moderation pattern differed by sport type. These findings may inform public health strategies that consider football-based activities as a promising context for adolescent mental health promotion.

## 1 Introduction

Adolescence is a critical period for identity formation and future mental health (1, 2), marked by rapid physiological and psychological changes (3, 4). Consequently, adolescence is a period of elevated risk for depression. According to the Report on National Mental Health Development in China (2021–2022), 14.8% of Chinese adolescents were identified as being at risk of depression, including 4.0% at risk of severe depression (5). Adolescent depression can lead to multiple negative outcomes. In the short term, it often results in academic impairment, social maladaptation, and a higher risk of substance abuse and suicide (6). In the long term, depression substantially increases the likelihood of developing mental disorders in adulthood (7). It is also associated with poor work performance, reduced income, suicidal ideation, and deteriorating physical health (4). Thus, adolescent depression is an important public health concern. Its substantial disease burden highlights the need for prevention and early intervention (8).

Upward social comparison may constitute a key risk factor for depression in adolescents (9, 10). Social comparison refers to the psychological process by which individuals evaluate their circumstances relative to others (11). Upward social comparison, a high-risk form, occurs when individuals compare themselves with others in superior positions (12). Social rank theory suggests that upward comparison may trigger a cycle of defeat and self-denigration, leading to depression (13). Adolescents, being in a sensitive stage of self-concept development, are especially prone to upward comparison (14). Their vulnerability stems from developmental factors: dependence on external validation during identity formation (15), repeated exposure to uncontrollable negative events fostering learned helplessness (16), low self-esteem (17), perfectionism-related stress (18), and immature cognitive control due to delayed prefrontal development (19). These combined factors constrain emotion regulation and promote rumination (20).

Sport participation may moderate the association between upward social comparison and depression. The Stress Buffering Hypothesis supports this perspective, positing that supportive external resources buffer against stress by mitigating maladaptive cognitions and regulating neuroendocrine responses, which in turn reduce vulnerability to depression (21). Sport participation fosters shared goals and collaboration, enabling adolescents to build deeper connections with peers and teachers, thereby enhancing social support and the ability to cope with stress (22, 23). Furthermore, the structured nature of interactions in sport, requiring frequent communication, conflict resolution, mutual encouragement, and emotional regulation, serves as a critical pathway for improving emotion regulation (24). Thus, sport participation provides alternative cognitive frameworks that counteract self-denigration arising from upward comparison. It also fosters supportive networks, such as peer encouragement and coach feedback. These social resources may help buffer adolescents against emotional exhaustion (25). These proposed processes provide the theoretical rationale for examining sport participation as a potential moderator of the association between upward social comparison and depression.

However, the moderation pattern may differ across types of sport participation. The Stress Buffering Hypothesis highlights that the efficacy of such buffering processes depends more on support quality than quantity (21). Evidence suggests that team sports may offer some advantages over individual sports in promoting adolescent mental health (26, 27). This advantage may stem from the social nature and positive experiences of team sports, such as stronger social support and an enhanced sense of belonging (28). From a macro perspective, team sports foster shared responsibility and collective identity, shifting self-evaluation from individual performance toward team contribution (29). Football has attracted growing interest as a context for adolescent participation and mental health promotion (30, 31). Recent research on youth soccer training has examined enjoyment as an experiential aspect of participation (32). Previous studies have suggested that greater engagement in football is associated with higher levels of psychological well-being (30). Such positive experiences may reduce adolescents’ tendency to interpret upward social comparison as evidence of inadequate personal ability. Participation in football may not only promote social skills and community belonging but also facilitate the development of effective communication and coordination, which are essential for overall development. More importantly, football’s distinctive team-based interaction structure may facilitate adolescents’ interpersonal relationships through continuous interactions with peers and coaches, including verbal encouragement during football activities (33, 34). This interaction structure expands access to social support and enables healthy development within a warm, supportive team climate (33). These social and team-based characteristics may provide adolescents with alternative self-evaluative frameworks and supportive interpersonal resources, thereby potentially weakening the process through which upward social comparison is translated into depressive symptoms. These characteristics may help explain why football, as a prototypical team sport, may show a different moderation pattern from that observed among participants in individual sports.

Although previous studies have examined the relationship between upward social comparison and depression, whether sport participation moderates this relationship and whether the moderation pattern differs by sport type remain unclear. To address this gap, this study, based on social rank theory and the Stress Buffering Hypothesis, constructed a moderation model (Figure 1) and proposed three hypotheses:

**Figure 1:**
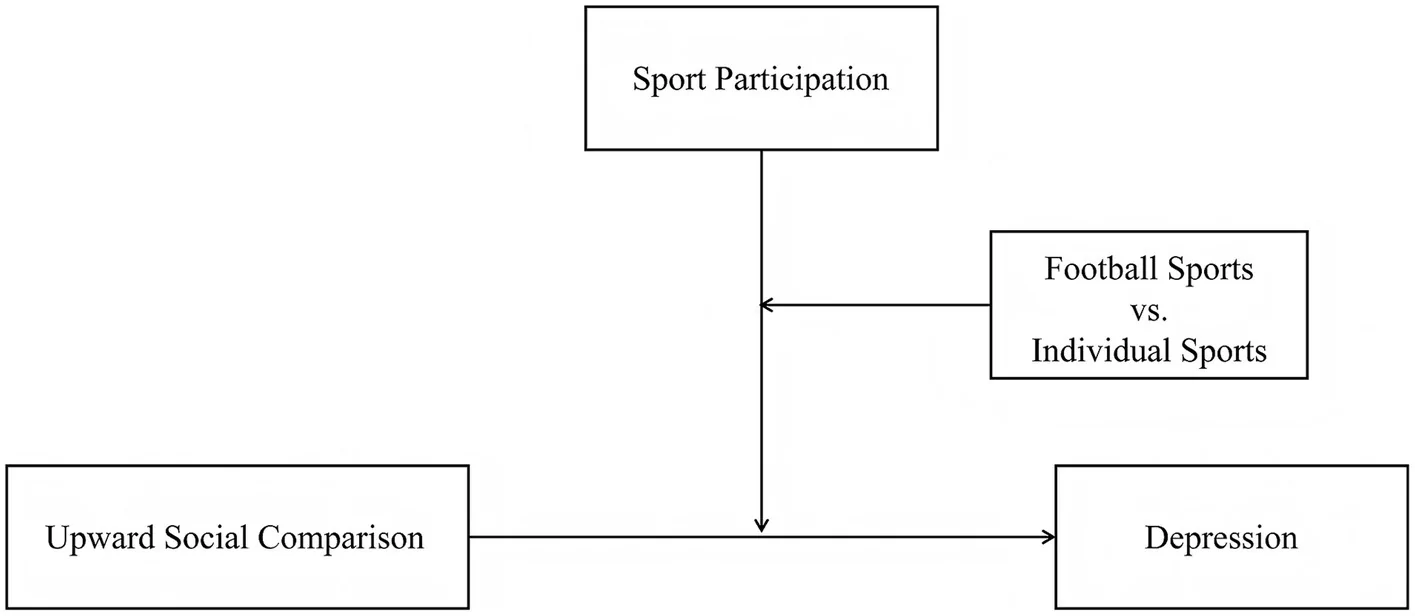
Moderation model illustrating the association between upward social comparison and depression, with sport participation and sport type (football vs. individual sports) as moderators. Control variables are omitted for clarity.

*H1*. Adolescents with higher levels of upward social comparison would report greater depressive symptoms.

*H2*. Higher sport participation would be associated with lower depressive symptoms and be associated with a weaker association between upward social comparison and depressive symptoms.

*H3*. The moderation pattern of sport participation in the association between upward social comparison and depressive symptoms would differ between football participants and participants in individual sports, with a potentially stronger moderation pattern in the football group.

## 2 Methods

### 2.1 Participants and procedure

This cross-sectional study adopted a purposive sampling method. The participating provinces were selected according to China’s seven major geographic regions. The survey covered 162 schools across 13 provinces, including Beijing, Inner Mongolia, Guangdong, Hainan, Hubei, Liaoning, Jilin, Shaanxi, Qinghai, Shandong, Fujian, Sichuan, and Yunnan. Within the participating schools, cluster sampling was used to recruit students, with classes serving as the sampling clusters. After obtaining written informed consent from all participants and their legal guardians, questionnaires were uniformly distributed within the primary and secondary school classes of the participating schools. After data collection, the responses were de-identified before being entered into the analytic dataset to protect participant anonymity. A total of 2,451 adolescents (age, *M* = 12.36, *SD* = 3.42) were initially recruited, including 1,516 football participants and 935 participants in individual sports. After excluding 16 cases because of incomplete responses, patterned answering, extreme response times, or failure to meet the sport-type grouping criteria (6 from the football group and 10 from the individual-sport group), 2,435 participants were included in the final analysis, yielding a validity rate of 99.4%. Inclusion criteria were as follows: (1) students enrolled in primary, junior high, or senior high schools; (2) students who were able to understand and complete the questionnaire independently; (3) students who had formal football training experience or participated exclusively in individual sports without participating in team sports or receiving formal football training; and (4) students who provided informed consent, with consent also obtained from their legal guardians. The analytic sample comprised 1,510 football participants and 925 participants in individual sports. Participants were classified into the football group or the individual-sport group according to their reported sport participation. The individual-sport group comprised 925 participants (38.0% of the final sample) who reported participation exclusively in individual sports and no participation in team sports and formal football training.

### 2.2 Measurement

#### 2.2.1 General information

The general information questionnaire collected demographic data, including gender (1 = male, 2 = female), school level (1 = primary school, 2 = junior high school, 3 = senior high school), and sport type (1 = football, 2 = individual sport). Prior research shows that demographic factors, including gender and school level, can affect the associations between football and mental health (35). Therefore, gender and school level were included as covariates. School level was selected as the indicator of educational stage because it reflects both developmental stage and the educational context of this school-based sample.

#### 2.2.2 Upward social comparison subscale (USC)

This study employed the Upward Social Comparison Subscale developed by Gibbons and Buunk (36, 37). The Chinese version has demonstrated good reliability and validity (38). The subscale consists of six items rated on a five-point Likert scale (1 = strongly disagree, 5 = strongly agree). In this study, the Cronbach’s *α* was 0.894.

#### 2.2.3 Beck depression inventory-II (BDI-II)

The Beck Depression Inventory-II (BDI-II) was employed in this study (39). The Chinese version has demonstrated good reliability and validity (40). The original version consists of 21 items, with each item rated on a 4-point scale from 0 to 3. Considering the characteristics of the adolescent sample in this study, and following expert panel review as well as school regulations concerning sensitive personal content, items 9 and 21 were removed. The final version therefore contained 19 items, with a possible total score ranging from 0 to 57, with higher scores indicating more severe depressive symptoms. In this study, the modified BDI-II was used solely as a continuous, dimensional measure of depressive symptoms; no clinical cut-off scores were applied, and the instrument was not used to establish clinical diagnoses. Confirmatory factor analysis was conducted based on the three-factor structure of the BDI-II reported in previous adolescent research (41). The model showed acceptable fit: *CFI* = 0.925, *TLI* = 0.913, *RMSEA* = 0.074, and *SRMR* = 0.043. The modified 19-item version also showed good internal consistency in the present sample (Cronbach’s α = 0.934), suggesting that it retained acceptable psychometric properties after item removal.

#### 2.2.4 Sports participation scale (SPS)

This study employed the Sports Participation Scale originally developed by Agbuga et al. (42). The Chinese version has demonstrated good reliability and validity in previous studies (43). The scale comprises 13 items across three dimensions: behavioral participation (Items 1–4), emotional participation (Items 5–8), and cognitive participation (Items 9–13). Items were rated on a five-point Likert scale and reverse-coded according to the original scoring procedure. The 13 items were then summed to generate a total sports participation score, with possible scores ranging from 13 to 65. Higher scores indicated greater sports participation. In this study, the Cronbach’s α was 0.986.

### 2.3 Data analysis

Data were analyzed using SPSS 27.0 and PROCESS version 4.2 (44). All analyses were conducted using a de-identified dataset containing individual-level records. Continuous predictors were mean-centered before the moderation analyses (45). First, SPSS was used to perform descriptive statistics, including the mean, standard deviation, and frequency distribution, for the demographic variables, as well as Pearson correlation analyses among the variables. Gender and school level were included as covariates in all moderation models to account for demographic and educational-stage differences. This study first used PROCESS Model 1 to test the moderation effect of sport participation on the relationship between upward social comparison and depression. This study then used PROCESS Model 3 to test whether this relationship was further moderated by sport type (football vs. individual sports). For all moderation analyses, simple slopes were probed at one standard deviation below and above the mean of sport participation, corresponding to 28.68 (*M* − 1 *SD*) and 52.99 (*M* + 1 *SD*), respectively.

## 3 Results

### 3.1 Basic characteristics

A total of 2,451 adolescents from primary school to senior high school participated in the study. Finally, 2,435 participants completed the full survey without missing responses, yielding a validity rate of 99.4%. Cases with incomplete responses, patterned answering, or extreme response times were excluded. Among the valid participants, 1,510 were football participants and 925 were participants in individual sports. The football group included 1,177 males and 333 females, and the individual-sport group included 632 males and 293 females. The distribution of school level did not differ significantly between the football and individual-sport groups, *χ*^2^(2) = 4.009, *p* = 0.135. However, the gender distribution differed significantly between the groups, *χ*^2^(1) = 27.810, *p* < 0.001. The detailed characteristics of participants are shown in Table 1.

**Table 1:** Demographic information of participants.

Variables	Total sample (*N* = 2,435)	Football participants (*n* = 1,510)	participants in individual sports (*n* = 925)	*X* ^2^	*p*
Gender				27.810	<0.001
Male	1,809 (74.3%)	1,177 (77.9%)	632 (68.3%)		
Female	626 (25.7%)	333 (22.1%)	293 (31.7%)		
Level of school				4.009	0.135
Primary school students	1,352 (55.5%)	836 (55.4%)	516 (55.8%)		
Junior high school students	666 (27.4%)	399 (26.4%)	267 (28.9%)		
Senior high school students	417 (17.1%)	275 (18.2%)	142 (15.4%)		

Pearson’ s chi-square tests were used to compare the football and individual-sport groups.

### 3.2 Assessment of common method bias

Harman’s single-factor test and an unmeasured latent method construct analysis were conducted to examine common method bias (46, 47). In Harman’s single-factor test, five factors with eigenvalues greater than 1 were extracted, and the first factor accounted for 32.785% of the variance, which was below the critical threshold of 40%. In the unmeasured latent method construct analysis, adding a common method factor produced only a small improvement in model fit compared with the baseline measurement model (*ΔCFI* = 0.002, *ΔTLI* = 0.002, *ΔRMSEA* = −0.001), indicating that common method bias was not a serious concern in this study.

### 3.3 Descriptive statistics and correlation analysis

Descriptive statistics and Pearson correlations among the variables are shown in Table 2. For the total sample (*N* = 2,435), sport participation was negatively correlated with depression (*p* < 0.001) and upward social comparison (*p* < 0.01), while upward social comparison was positively correlated with depression (*p* < 0.001).

**Table 2:** Descriptive statistics and correlation analysis (*N* = 2,435).

Variables	*M*	SD	1	2	3
Sport Participation	40.840	12.159	1		
Depression	24.300	8.317	−0.172***	1	
Upward Social Comparison	19.930	6.320	−0.062**	0.090***	1

The mean (M) and standard deviation (SD). *p < 0.05, **p < 0.01, ***p < 0.001.

### 3.4 Moderation effect

After controlling for gender and school level, moderation analyses were conducted using Hayes’ PROCESS Model 1 (44), with upward social comparison as the independent variable, depression as the dependent variable, and sport participation as the moderator. Results are reported in Table 3. Upward social comparison was positively associated with depression (*b* = 0.079, *SE* = 0.020, *t* = 3.904, *p* < 0.001), indicating that higher levels of upward comparison were associated with more severe depressive symptoms. Sport participation was negatively associated with depression (*b* = −0.155, *SE* = 0.021, *t* = −7.564, *p* < 0.001), suggesting that greater sport participation was associated with lower depression. The interaction between upward social comparison and sport participation was significant (*b* = −0.055, *SE* = 0.019, *t* = −2.821, *p* = 0.005), indicating that sport participation was associated with a weaker positive association between upward social comparison and depression. The interaction term explained an additional 0.32% of the variance in depression (*ΔR^2^* = 0.003), indicating a small incremental contribution.

**Table 3:** Simple moderation analysis (*N* = 2,435).

Variables	Coefficient	SE	*t*	LLCI	ULCI
Upward Social Comparison (X)	0.079***	0.020	3.904	0.039	0.119
Sport Participation (W)	−0.155***	0.021	−7.564	−0.196	−0.115
X × W	−0.055**	0.019	−2.821	−0.092	−0.017
Constant	−0.338***	0.074	−4.581	−0.483	−0.193
Gender	0.061	0.046	1.324	−0.029	0.151
School Level	0.159***	0.026	6.101	0.108	0.211
Model summary					
*R*	0.235	*R^2^*	0.055	MSE	0.947
*F*(5, 2,429)	28.437***	*ΔR^2^* (X × W)	0.003	*ΔF*(1, 2,429)	7.960
				*P* for *ΔF*	0.005

Gender and school level were included as covariates. Analyses were conducted using individual-level records. LLCI, lower limit of the 95% confidence interval; ULCI, upper limit of the 95% confidence interval. *p < 0.05, **p < 0.01, ***p < 0.001.

Simple slope analysis (Figure 2) showed that, for adolescents with low sport participation (*M* – 1*SD*), upward social comparison was significantly associated with depression (*simple slope* = 0.133, *t* = 4.557, *p* < 0.001, 95% CI [0.076, 0.190]). For adolescents with high sport participation (*M* + 1 *SD*), this association was weaker and not significant (*simple slope* = 0.038, *t* = 1.601, *p* = 0.110, 95% CI [−0.009, 0.085]).

**Figure 2:**
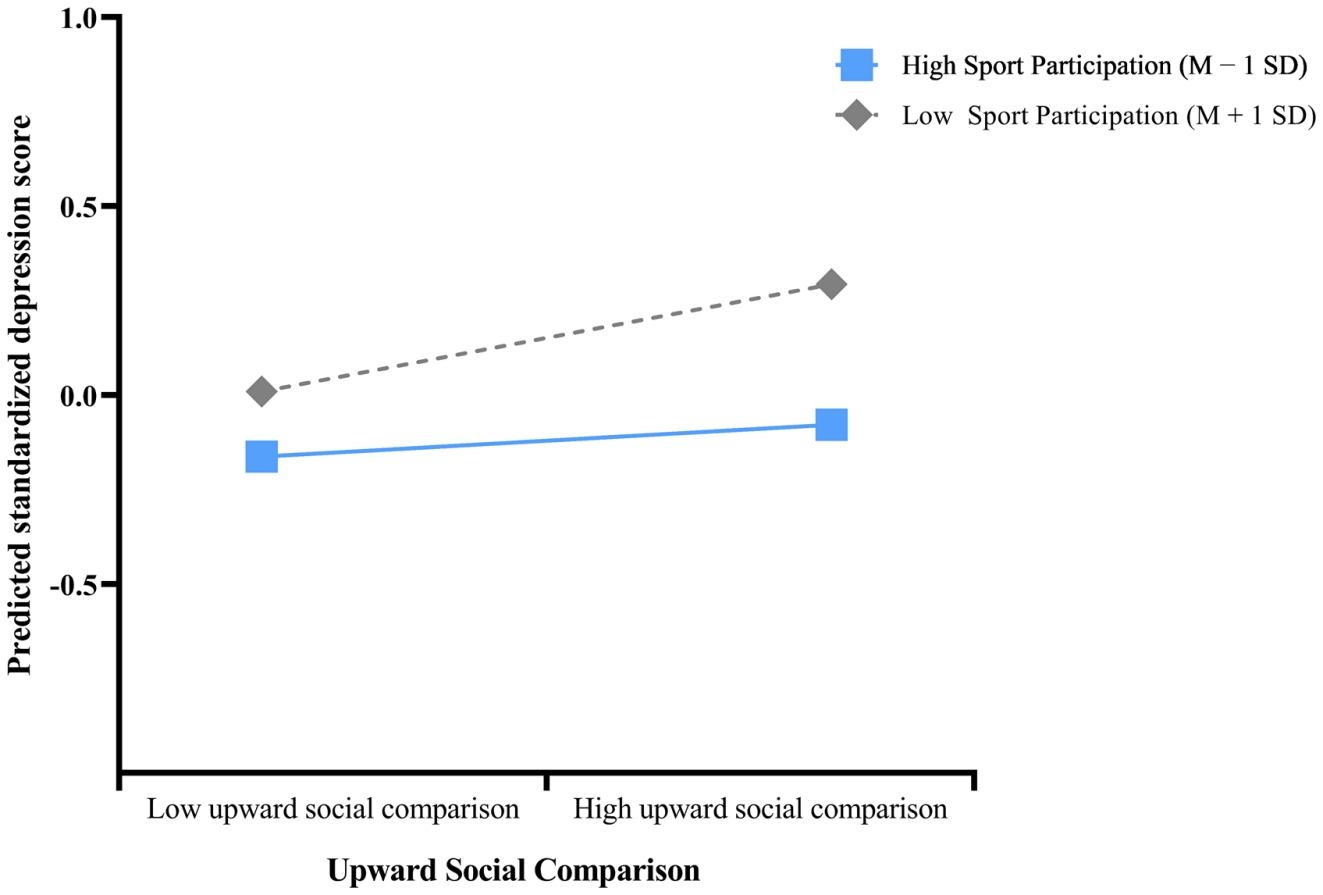
Simple-slope analysis showing the moderation of the association between upward social comparison and depression by sport participation. Low and high sport participation correspond to *M* − 1 *SD* and *M* + 1 *SD*, respectively. The *y*-axis shows predicted standardized depression scores.

Furthermore, after controlling for gender and school level, a three-way interaction analysis tested whether sport type moderated the interaction between upward social comparison and sport participation. Results (Table 4) showed that the interaction among upward social comparison, sport participation, and sport type was significant (*b* = 0.163, *SE* = 0.053, *t* = 3.096, *p* = 0.002, 95% CI [0.060, 0.266]). This three-way interaction explained an additional 0.38% of the variance in depression (*ΔR^2^* = 0.004), also representing a small incremental contribution.

**Table 4:** Three-way interaction analysis of upward social comparison, sport participation, and sport type in predicting depression (*N* = 2,435).

Variables	Coefficient	SE	*t*	LLCI	ULCI
Upward Social Comparison (X)	0.066**	0.021	3.162	0.025	0.107
Sport Participation (W)	−0.187***	0.023	−8.251	−0.232	−0.143
Sport Type (Z)	−0.060	0.042	−1.412	−0.142	0.023
X × W	−0.017	0.023	−0.761	−0.062	0.027
X × Z	−0.142**	0.044	−3.237	−0.228	−0.056
W × Z	−0.243***	0.051	−4.735	−0.343	−0.142
X × W × Z	0.163**	0.053	3.096	0.060	0.266
Constant	−0.330***	0.074	−4.485	−0.474	−0.186
Gender	0.059	0.046	1.281	−0.031	0.149
School Level	0.164***	0.026	6.328	0.113	0.215
Model summary					
*R*	0.268	*R^2^*	0.072	MSE	0.932
*F*(9, 2,425)	20.780***	ΔR^2^ (X × W × Z)	0.004	*ΔF*(1, 2,425)	9.587
				*P* for *ΔF*	0.002

Gender and school level were included as covariates. Analyses were conducted using individual-level records. LLCI, lower limit of the 95% confidence interval; ULCI, upper limit of the 95% confidence interval. *p < 0.05, **p < 0.01, ***p < 0.001.

As illustrated in Figure 3, among football participants, upward social comparison was significantly associated with depression at low levels of sport participation (*M* − 1 *SD*, *simple slope* = 0.201, *t* = 6.130, *p* < 0.001, 95% CI [0.137, 0.265]). At high levels of sport participation (*M* + 1 *SD*), this association was weaker but remained significant (*simple slope* = 0.061, *t* = 1.991, *p* = 0.047, 95% CI [0.001, 0.121]). By contrast, among participants in individual sports, upward social comparison was not significantly associated with depression at either low levels of sport participation (*M* − 1 *SD*, *simple slope* = −0.103, *t* = −1.451, *p* = 0.147, 95% CI [−0.243, 0.036]) or high levels of sport participation (*M* + 1 *SD, simple slope* = 0.041, *t* = 1.073, *p* = 0.283, 95% CI [−0.034, 0.116]). Taken together, these findings indicate that the conditional association between upward social comparison and depression differed according to sport type and level of sport participation. In the football group, the association was weaker at higher levels of sport participation but remained statistically significant, whereas no significant conditional association was observed in the individual-sport group.

**Figure 3:**
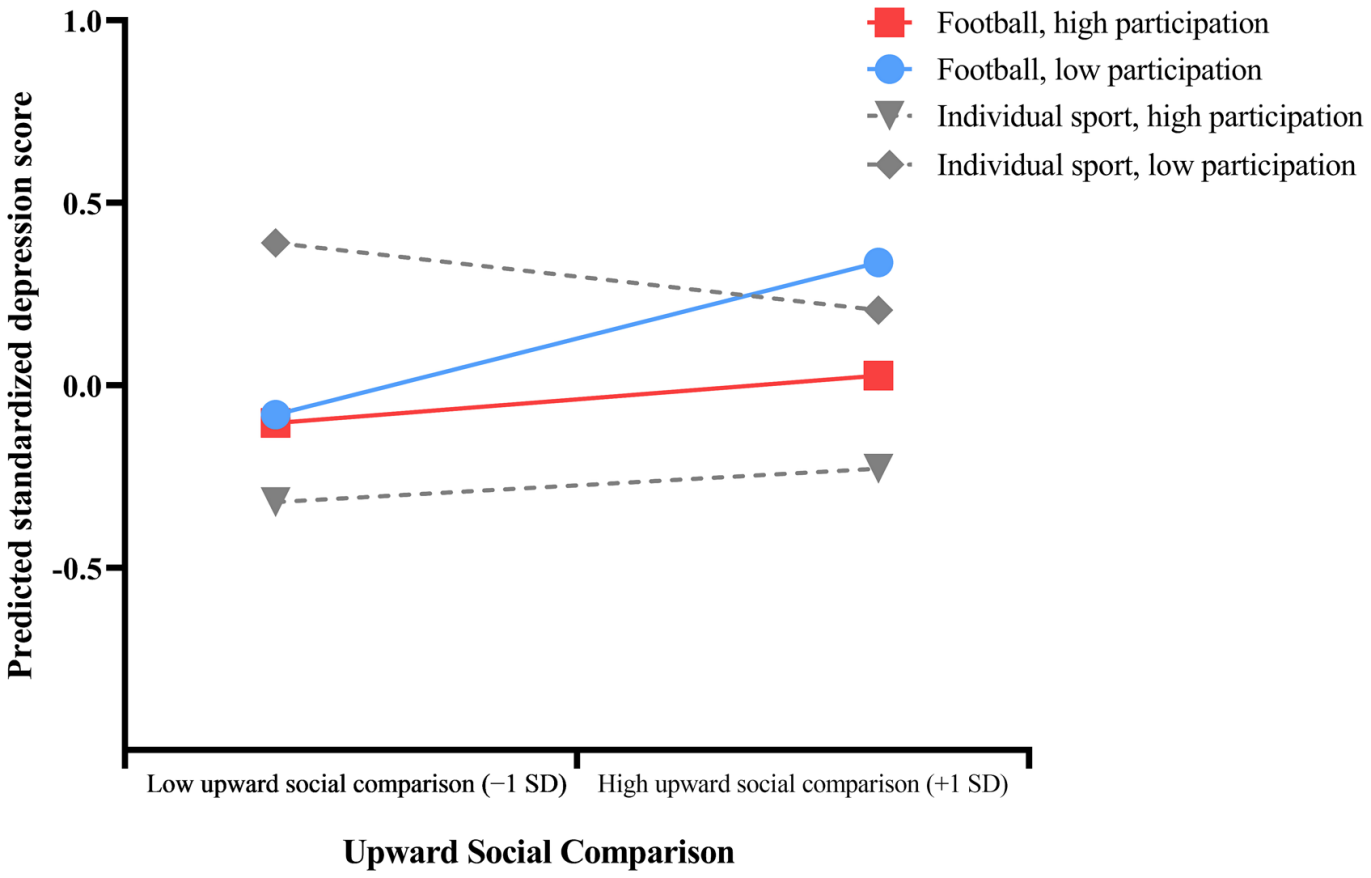
Three-way interaction among upward social comparison, sport participation, and sport type. Low and high sport participation correspond to *M* − 1 *SD* and *M* + 1 *SD*, respectively. The moderation pattern differed between football participants and participants in individual sports. The *y*-axis shows predicted standardized depression scores.

## 4 Discussion

Building on social rank theory, the Stress Buffering Hypothesis, and previous research, this study constructed two analytical models. The results yielded three key findings: (1) upward social comparison was positively associated with depression; (2) sport participation was associated with a weaker positive association in the overall sample; (3) the moderation pattern differed between football participants and participants in individual sports. These findings may support the consideration of football-based activities as a promising context for adolescent mental health promotion within public health. Although both interaction terms were statistically significant, their incremental contributions to the explained variance were small. Because interaction effects are often modest in magnitude and may require relatively large samples to detect, the large sample size in the present study may have increased the sensitivity of the analyses to these small effects (48, 49). Thus, the statistical significance of these interactions should be interpreted alongside their small incremental contributions. Because potential within-school dependence was not modeled, the statistical significance and robustness of these modest effects should also be interpreted cautiously. These findings provide an initial empirical basis for future longitudinal and intervention studies to evaluate their practical and clinical relevance.

Consistent with H1, this study found that upward social comparison was positively associated with depressive symptoms, in line with previous research and with social rank theory (13, 38). Social rank theory explains depression from the perspective of evolutionary psychology. According to this theory, perceptions of inferiority in social competition can activate defeat and submission responses, which may contribute to depression (13). During self-identity development, adolescents with trait anxiety and low self-acceptance are more likely to see the gap between their actual and ideal selves as fixed personal flaws rather than as changeable external conditions (14). Upward social comparison fosters persistent negative self-cognitions that make adolescents undervalue their abilities and, at the same time, elicits negative emotions such as envy and shame (50, 51). When combined with immature emotion regulation, these processes become central mechanisms of depression. At the neurobiological level, ongoing prefrontal development may leave adolescents more vulnerable to difficulties in evaluating stressful situations and regulating emotional responses (19, 52). Previous research has linked chronic stress and rumination during adolescence to alterations in cortisol regulation, hippocampal structure, and rumination-related neural activity (53–55). Together, this literature provides a broader neurobiological context for understanding how upward social comparison may be associated with depressive symptoms and suggests avenues for future mechanistic research.

This study found that sport participation was not only negatively associated with depression but also moderated the association between upward social comparison and depression, consistent with H2 and the Stress Buffering Hypothesis (21). This hypothesis posits that supportive external resources buffer stress responses and reduce the harmful impact of maladaptive cognitions on mental health. From a psychological perspective, sport participation may alter adolescents’ evaluative standards. It shifts self-assessment away from external appearance and toward ability and performance. This transition decreases body-image concerns and promotes self-esteem (56, 57). Elevated self-esteem provides a cognitive and emotional buffer. It may weaken the association between upward social comparison and depressive symptoms. From a social perspective, sport offers adolescents structured opportunities for peer and coach interactions. Through collective training, they receive encouragement from peers and support from coaches (58). Interpersonal behaviors in sport settings may further shape athletes’ psychological experiences and motivation (59). These experiences foster positive feedback loops. Such external reinforcements may compensate for reduced self-efficacy and counteract the internalization of negative self-appraisals (60). At a broader neurobiological level, regular physical activity has been associated with improvements in mood, cognition, and emotion regulation (61, 62). This may be relevant to the lower depressive symptoms and weaker association between upward social comparison and depression observed among adolescents with higher levels of sport participation.

Consistent with H3, this study found that the moderation pattern of sport participation differed by sport type. Specifically, the three-way interaction among upward social comparison, sport participation, and sport type was significant. The interaction between upward social comparison and sport participation was significant among football participants but not among those engaged in individual sports. Thus, the present findings indicate different conditional association patterns between the two sport-type groups. This pattern is consistent with the Stress Buffering Hypothesis (21). The hypothesis posits that external supportive resources function as psychological buffers. Such resources are likely to depend on high-quality emotional bonds, frequent interactions, and functional interdependence. Football, as a socially intensive team sport, may provide such conditions. The cooperative framework of football requires participants to engage in frequent role negotiation and tactical coordination (63), which may shift self-evaluation from competing with others toward contributing to the team (64). Through role specialization, adolescents may derive self-worth not only from outperforming others but also from fulfilling specific roles and contributing to shared performance (65). By contrast, individual sports may offer fewer opportunities for high-quality emotional bonds and frequent interactions than team sports, which may contribute to different moderation patterns across sport-type groups (66). This difference may help explain why participation in individual sports did not show a comparable moderation pattern in the present study.

In addition, at the competitive level, individual sports usually follow a binary win-or-lose logic, whereas football can include draws and more varied performance outcomes. This evaluative structure can emphasize process rather than outcome and encourage adolescents to value enjoyment and effort over results (67, 68). Motivational processes, including intrinsic and extrinsic factors, may also shape athletes’ engagement and performance in football contexts (69). In this context, football may reduce performance-oriented pressure and be associated with a weaker negative emotional response to upward social comparison. By contrast, individual sports may place greater emphasis on personal performance and self-focused comparison. Although this may stimulate short-term motivation, it fosters habitual upward comparison and performance-based self-worth (70). Football also requires a high degree of interaction and interdependence. Participants must continuously adjust positions, pass, and coordinate defense to respond to dynamic game contexts (27, 71). Through dynamic collaboration, shared responsibility, and team cohesion, football may provide a socially dense context that supports emotional regulation and self-worth through structured cooperation (29, 33, 72, 73). Previous studies have suggested that football participation may involve neurocognitive demands related to decision-making and cognitive control (74, 75), providing a possible neurocognitive perspective for understanding football’s moderation pattern. However, these processes were not directly examined in the present study and should therefore be viewed as a theoretical basis for future football-based mental health promotion research.

These findings should also be understood in the context of Chinese adolescents. In China, school life is often highly structured and academically competitive, and adolescents may be frequently exposed to performance-based evaluation and social comparison (76, 77). Chinese culture also places strong emphasis on interdependence, group belonging, and collective goals, which differs from many Western cultural contexts (78). In this context, sport participation may provide an alternative setting for peer interaction, self-evaluation, and stress relief. Recent longitudinal evidence from Chinese adolescents also suggests that football engagement is associated with self-esteem and depression (35). Football, as a team-based activity, may be especially relevant because it emphasizes cooperation, role division, and shared goals (33, 63, 70). These team-based experiences may offer adolescents a source of recognition outside academic performance and individual ranking. They may also help adolescents evaluate themselves through contribution to the group rather than through upward comparison alone. These contextual features may help interpret the observed pattern and provide a useful basis for future cross-cultural research.

The findings of this study not only extend the theoretical framework explaining the link between social comparison and depression but also suggest potential directions for future research on adolescent mental health promotion. Although the observed interaction effects were modest in magnitude, they may provide an initial basis for considering how school-based mental health promotion programs could be developed and evaluated (79). At the educational level, football and other team sports may serve as potential contexts for future school-based interventions. Such interventions could examine whether teamwork, role division, and situational interaction contribute to healthier self-evaluations and fewer depressive symptoms among adolescents experiencing upward social comparison (80). At the public health level, team sports, including football, may be explored as complementary interventions within broader adolescent mental health promotion efforts. Future longitudinal and intervention studies should assess the feasibility and effectiveness of such activities, particularly among adolescents at high risk. Such evidence could help clarify their potential to complement traditional counseling by fostering motivation and social connectedness (81, 82).

This study also has several limitations. First, because the design was cross-sectional, temporal order and causality cannot be established (83). Thus, the observed moderation pattern should be interpreted as associative rather than causal. Future longitudinal studies are needed to examine the temporal relationships among upward social comparison, sport participation, sport type, and depression. Second, several measurement-related limitations should be noted. All constructs were assessed using self-report measures, which may introduce common method bias that cannot be fully ruled out even with statistical checks (47). In addition, although the modified 19-item BDI-II showed acceptable psychometric properties in the present sample, removing two items may limit its comparability with studies using the full 21-item BDI-II (84). Because the modified version was used only as a dimensional measure, no clinical cut-off scores were applied, and the cut-offs established for the original BDI-II cannot be assumed to be valid for the modified version. Future studies should combine self-report measures with multi-informant reports or objective indicators, and further examine the measurement equivalence, criterion validity, and clinical interpretability of the modified BDI-II with the full original version or other standardized depression measures. Third, although gender and school level were controlled, socioeconomic status, family support, and previous mental health history were not assessed, leaving potential residual confounding. Because sport type was not randomly assigned, selection bias may also have contributed to the observed group differences (85). The individual-sport group was an aggregated category restricted to participants with no team-sport participation or formal football training; however, the included activities may differ in their physical, psychological, and social characteristics, and sport-specific analyses were not possible. Therefore, the findings should be interpreted as a comparison between football participants and an aggregated individual-sport group, rather than as evidence concerning individual sports in general. Future studies should assess a broader range of demographic and psychosocial factors and use longitudinal, quasi-experimental, or propensity-score approaches to address potential confounding and selection bias. Fourth, the hierarchical structure of students nested within schools could not be accounted for because the de-identified analytic dataset did not retain school-level linkage identifiers. Consequently, school-level ICCs could not be estimated, and cluster-robust standard errors or multilevel models could not be applied. Within-school dependence may therefore have resulted in underestimated standard errors and affected statistical inference (86). Given the small incremental variance explained by the interaction terms, their statistical significance and robustness should be interpreted cautiously. Future studies should retain anonymized school-level identifiers and use cluster-robust or multilevel models to account for the nested data structure.

## 5 Conclusion

In conclusion, upward social comparison was positively associated with adolescent depression, and this cross-sectional association was weaker at higher levels of sport participation. This moderation pattern differed by sport type: among football participants, the association was weaker at higher levels of sport participation, whereas the corresponding pattern was not statistically supported among participants in individual sports. These findings suggest that sport type may be associated with different moderation patterns in the relationship between upward social comparison and depression. At the same time, they provide preliminary empirical evidence from Chinese adolescents that may inform public health considerations of sport-based mental health promotion and support further longitudinal and intervention research on football and other team sport settings.

## Data Availability

Data are available from the corresponding author upon reasonable request.
